# The Effects of Physical Activity Feedback on Behavior and Awareness in Employees: Study Protocol for a Randomized Controlled Trial

**DOI:** 10.1155/2012/460712

**Published:** 2012-09-25

**Authors:** Karen Van Hoye, Filip Boen, Johan Lefevre

**Affiliations:** Department of Kinesiology, Physical Activity, Sports and Health, Katholieke Universiteit Leuven, Tervuursevest 101, 3001 Leuven, Belgium

## Abstract

*Purpose*. The SenseWear Armband (SWA) is a multisensor activity monitor that can be used in daily life to assess an individual's physical activity level (PAL). The primary goal of this study was to analyze the impact of different types of feedback on the PAL of Flemish employees. *Methods/Design*. We recruited 320 sedentary employees (age, 41.0 ± 10.7 years; BMI, 26.2 ± 4.2 kg/m^2^) to participate in the 12-month study. Participants were randomized into one of four intervention groups after being measured for 7 days and nights by means of the SWA: (1) a minimal intervention group received no feedback (MIG, *n* = 56); (2) a pedometer group was provided only information on their daily step count (PG, *n* = 57); (3) a display group received feedback on calories burned, steps taken, and minutes of physical activity by means of the SWA display (DG, *n* = 57); (4) a coaching group also received the display and had weekly meetings with a Personal Coach (CoachG, *n* = 57). We hypothesize that participants receiving feedback (SG, DG, and CoachG) will have a greater increase in physical activity outcome variables compared to participants of the minimal intervention group.

## 1. Introduction

Increasing daily physical activity (PA) is a key strategy for preventing or minimizing numerous chronic diseases [1, 2]. Daily PA consists of all occupational, domestic, and leisure-time activities. While participation in leisure-time activities has remained relatively stable over the years, technological advancements have drastically reduced occupational PA [3]. People spent more time at sedentary occupations or in work-related activities such as passive commuting (e.g., driving/riding in a motorized vehicle), which has a negative impact on total PA [4]. 

Given the benefits of regular PA and the current high prevalence of physical inactivity, people need strategies to increase their level of PA [5]. In 1996, the American College of Sports Medicine proposed PA guidelines which stated that 30 minutes of moderate-intensity exercise on most days provide some health benefits, notably, a decreased risk of chronic diseases. Moderate intensity PA is defined as activity performed at 3–6 MET and corresponds with the equivalent of walking 3-4 mph for most healthy adults. Because inactive people show measurable health benefits with only small increases in activity levels, these people should be targeted in health-promotion programs [6].

 The physical activity level (PAL) of an individual is widely used as an indirect measure of physical activity energy expenditure. It can be measured or estimated from the average of 24-hour total energy expenditure (TEE) and the basal metabolic rate (BMR) (i.e., PAL = TEE/BMR). The expression of energy requirements of adults as PALs provides a convenient way of controlling for age, sex, weight, and body composition. A meta-analysis of studies that involved a total of 411 men and women from 18 to 64 years of age with a predominantly sedentary western lifestyle showed a mean value for PAL of 1.60 (range 1.55 to 1.65) for both men and women [7]. A PAL value of 1.75 and higher includes the regular practice of PA at work or in leisure time with an intensity and duration that will reduce the risk of becoming overweight and developing a variety of noncommunicable chronic diseases.

Research has revealed an association between a person's physical activity level and his/her lifestyle. According to the 1981 FAO/WHO/UNO expert consultation, an active or moderate active lifestyle corresponds with a PAL value between 1.70 and 1.99. According to this expert panel, the daily performance of one hour (either continuous or in several bouts during the day) of moderate to vigorous exercise, such as jogging/running, cycling, aerobic dancing, or various sports activities, can raise a person's average PAL from 1.55 (corresponding to the sedentary category) to 1.75 (the moderately active category). 

Inactive subjects are often not aware of the fact that they are insufficiently active. Physical activity awareness—defined as the agreement between self-rated and actual activity level according to current guidelines—has rarely been studied as a determinant of healthy behavior change. People are only expected to consider changing their behavior when they become aware that they personally engage in too little PA and are potentially putting their health at risk [8]. One way of raising awareness of individual physical (in)activity levels compared with perceived (in)activity levels is to monitor their physical (in)activity, for instance, by using objective measures such as a pedometer or a multisensor activity monitor [9].

Several studies have promoted pedometers as effective tools to increase walking [10, 11]. The 10,000 steps/day program has been the most popular proposal [12]. Pedometers measure walking through step counts and have been proven to be both reliable and valid [13, 14]. Most of the research on pedometers has focused on their use as a measurement device for assessing PA and not as a motivational tool [15]. Recently, studies examining the effectiveness of pedometers in promoting PA have shown that PA programs consisting of a pedometer, a daily goal defined in steps, and a step diary will increase physical activity levels by 2500 steps on average, compared with a sedentary control group [16–18]. It therefore has been concluded that pedometers can motivate individuals by monitoring the step count and operate as a tracking device and goal setting tool [19].

Although pedometers are easy-to-use motivational instruments, their major drawbacks are that they cannot reflect intensity of movement and therefore result in inaccurate energy expenditure estimations [20]. By contrast, the SenseWear Pro3 Armband (SWA) (BodyMedia, Inc. Pittsburgh, PA, USA) is a multisensory device that is worn on the upper arm and receives information from a dual-axial accelerometer to measure motion and multiple sensors to measure skin temperature, heat flux, and galvanic skin response. The SWA also works with a watch-like display device that gives the user real-time feedback on calories burned, steps taken, and time spent in various intensity zones. Time spent in moderate, vigorous, and very vigorous activities corresponds with MET values greater than or equal to 3 and less than 6, greater than or equal to 6, and greater than or less than 9, respectively. Such a sophisticated accelerometer-based device has advantages over pedometers when energy-related output variables are a desired outcome. When people are given information on the energy expenditure (kilocalories) and the time spent (minutes) of physical activities above a predetermined intensity level as well as the number of steps, they will have a very detailed description of their own physical (in)activity pattern. To our knowledge, no study has so far investigated whether specific feedback on various dimensions of the physical (in)activity behavior leads to a greater increase of the individual's physical activity level compared with more basic feedback on their number of steps.

When people are being measured, they become more self-conscious about their own behavior. It is well documented that promoting PA using simple, attainable behavioral strategies such as self-monitoring, goal setting, and physical (in)activity behavior feedback is effective for interventions [21]. Self-monitoring increases awareness of energy expenditure, enhances self-efficacy, allows for individuals to monitor progress and change over time, and thus can be an effective tool for behavioral change. Goal setting has shown some promise in promoting dietary and PA behavior change among adults [22]. Cullen et al. [23] developed a four-step goal-setting process which consists of (1) recognizing a need for change, (2) establishing a goal, (3) adopting a goal-directed activity and self-monitoring it, and (4) self-rewarding goal attainment. Adding feedback and rewards to the goal-setting process can increase motivation and task performance, and ultimately this leads to behavior change. Feedback can be described as knowledge of personal status about one's selected goal and should be provided regularly to adapt or adjust to the required behavior [24]. Information from a PA monitor can help the user in attaining their exercise goals. The simple retargeting of information about the physical (in)activity behavior to the individual can have immediate impact on their exercise behavior. Paschali et al. [25] used simple accelerometers to track activity among adults with type 2 diabetes. This pilot study examined whether giving activity feedback to obese, sedentary adults with type 2 diabetes would improve their adherence to a home-based walking program. Since the authors could not significantly contribute the increase in PA to the feedback condition, they concluded that further studies with larger sample sizes and greater control of experimental conditions were needed to determine the utility of objective activity feedback.

The SenseWear display is a recent innovation which allows progress monitoring throughout the day and indicates in real time when goals have been met. This technology can help motivate people towards predetermined activity goals allowing them to have a clearer picture of their physical (in)activity pattern. Setting specific goals is an important aspect of coaching as the effectiveness of coaching revolves around the nature of the goals established by the individual and the processes by which they are achieved. Coaching is a behavioral strategy recently emerging in the literature and works as an effective strategy in achieving lifestyle change [26]. The social interactions provided by a coach may provide useful forms of modeling and social support that can enhance the initial adoption of health behaviors such as PA [27]. Understanding the factors that can motivate health-enhancing PA has considerable value given the role of this lifestyle behavior in promoting quality of life. One theoretical perspective that appears useful for understanding motivation and adherence to exercise is the self determination theory [28]. SDT particularly describes the processes through which a person acquires the motivation for initiating new health-related behaviors and maintaining them over time. The theory focuses on the degree to which people's motivation toward engagement in activities, such as PA, is more or less self-determined (autonomous) or controlled by external or internal pressures [29]. People are autonomously motivated when they engage in an activity or cease an activity for reasons that are freely chosen. In a PA context, people are autonomously motivated if they choose to initiate PA for enjoyment and/or because they think PA is important and will help them attain valued goals and/or because of personal commitment to improving their health or quality of life. Research has shown that autonomous motivation leads to greater long-term persistence, for example, maintained change toward healthier behaviors [30].

SDT proposes that humans have three basic psychological needs of autonomy, competence, and relatedness that must be satisfied in order for growth and well-being to be achieved [31]. Autonomy relates to the desire to be the regulator of one's actions, and that behavior is volitional. People perceive themselves to be competent when they feel capable of attaining health outcomes, such as meeting a PA goal. The construct of perceived competence is very similar to the self-efficacy concept [32, 33], which has been found to be one of the strongest predictors of PA in adults [34]. Relatedness refers to experiencing care and concern from important individuals and feeling connected and understood by others [35]. SDT argues that developing a sense of autonomy and competence is critical to self-regulate and sustain health-promoting behavior. Equally important is relatedness, as people are more likely to adopt behaviors promoted by those whom they trust [36].

Over the years, the number of social factors that have been found to affect people's needs and, in turn, motivation has been growing [37]. How other people behave has tremendous impact on the needs being satisfied and, in turn, on motivation being optimal or not. One of the key interpersonal dimensions studied is autonomy support which can be defined as the active support of the person's capacity to be self-initiating and autonomous [38]. Autonomous supportive environments can be established by acknowledging the feelings and perspectives of the individual, by providing sufficient information, by minimizing pressure and control, and by offering freedom of choice [38]. 

SDT has been utilized with a variety of health behaviors. Silva et al. [39] implemented an SDT-based intervention for weight management, facilitating exercise adherence by enhancing the more autonomous forms of behavioral regulation. Patrick and Canevello [40] used a computerized intervention based on SDT to better understand the psychological mechanisms in regard to PA frequency, intensity, and duration in sedentary young adults. These studies highlight the positive influence that autonomy support can have on facilitating health behavior change as well as associated physical and psychological health benefits [41]. 

Well-designed randomized controlled trials (RCTs) could help in understanding what types of interventions promote change in a particular behavior. Research shows that individually tailored interventions are more likely to be read, saved, remembered, and discussed [42], and that goal setting in combination with self-monitoring is more successful [27, 43]. We hypothesize that the use of the feedback generated by a SenseWear display or a pedometer will increase awareness of employees own physical (in)activity pattern and feelings of competence and results in an increased PAL in comparison with a minimal intervention group.

For motivational purposes, a simple, low-cost step counter may suffice, and these are more feasible from a cost-efficiency perspective than sophisticated accelerometer-based devices [44]. Because the SWA display gives continuous feedback about people's (in)activity behavior and specific activity goals can be set, research is needed to compare the individual's adherence with a healthy lifestyle when feedback is given by a pedometer or a multisensor activity monitor. This study has the additional purpose to compare the effectiveness of feedback given by a pedometer and a multisensor device. We hypothesize that activity monitors that display specific feedback on several parameters of an individual's activity pattern, namely, steps, calories, and minutes spent on PA will motivate people to increase their activity level more than monitors who only track step counts.

The coaching is based on the SDT of Deci and Ryan. This theory states that the extent that the environment allows one to experience feelings of competence, autonomy, and relatedness will influence the person's motivation toward a given task. The behavior of a coach has a strong impact on people's needs being satisfied and, in turn, on their motivation being optimal or not. It can be speculated that if the relation with a coach leads to relatedness satisfaction, the message and values transmitted by the coach may be more readily internalized by the participant. Furthermore, to the extent that the coach also supports the client's autonomy, such internalization will be autonomous in nature, thereby leading to positive and long-lasting PA changes in the participant. Our intervention study wants to determine the effectiveness of continuous self-monitoring and real-time feedback from the SenseWear Armband alone and in combination with a weekly meeting with a personal coach. We hypothesize that the weekly meeting with a coach will help create an autonomous supportive environment and stimulate the feeling of relatedness. 

## 2. Methods/Design

### 2.1. Study Design

We performed a 12-month randomized controlled trial in which we randomly assigned sedentary male (*n* = 103) and female (*n* = 124) employees to one of four intervention groups: a minimal intervention group received no feedback during the intervention period (MIG; *n* = 56); a pedometer group received information on their daily step count and received a step diary to write down their daily steps (PG; *n* = 57); a display group got personalized feedback by means of the SWA display and received a diary to write done their additional activities (DG; *n* = 57); a coaching group also received the display and diary and additionally had weekly meetings with a coach to discuss the progress they had made (CoachG; *n* = 57). During those meetings, the coach attempted to create an autonomy-supportive environment by encouraging choice and initiation (“how would you increase your activities this week?”), providing participants with different options (“maybe you could go for a walk during your lunch break or travel to work by bike?”), giving informational positive feedback, and acknowledging that the feeling of competence grows from feedback inherent to task (“well done, this week you participated at least more than one hour a day in PA”).

Outcome measures were assessed during a baseline assessment and again at 3, 6, and 12 months. The primary outcome of the study is the level of physical activity (PAL). Secondary outcome measures include step count, minutes of physical (in)activity (sedentary, light, moderate, vigorous, and very vigorous intensity PA), daily energy expenditure in PA, percentage of participants losing fat percentage, and stages of motivational readiness for PA.

### 2.2. Description and Selection Criteria of Participants

All participants were recruited from working places in Flanders (Belgium) through flyers, emails, pharmacists, and word of mouth. Employees (male and female) aged between 19 and 67 years who mentioned not being physically active during the last year, who are interested in knowing how many calories they burn in daily life, and who are motivated wearing the SenseWear Armband during 6 weeks were recruited. 

### 2.3. Participant Screening

**Figure 1:**
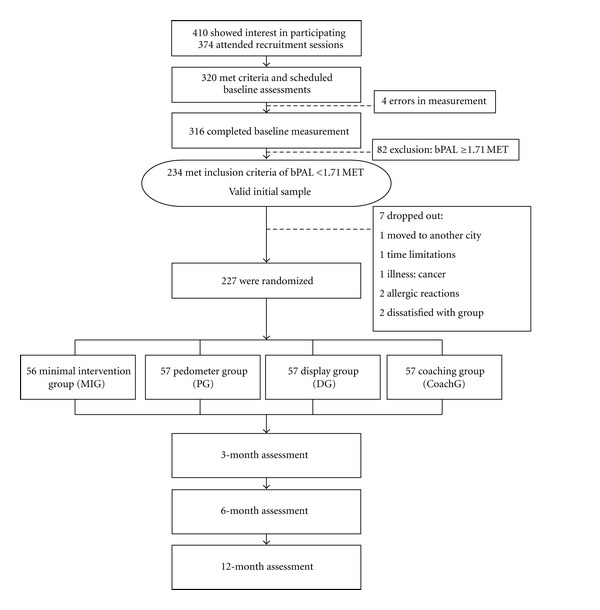
Participants flow from screening to randomization. All four groups received the SWA during the intervention period and the posttests. The minimal intervention group received no feedback during the 4-week intervention period. The pedometer group received feedback on the daily step count and a step diary during the 4-week intervention period. The display group and coaching group received a SenseWear display and an activity diary during the 4-week intervention period.

Participants' flow of the study is shown in Figure 1. All study participants progressed through the study, beginning with an initial contact in which participants are invited to go to an information session. At this session, potential participants received an explanation regarding the purpose of the study and study expectations. This discussion included the participant responsibilities and potential health benefits and risks related to study participation. All interested participants had to sign an informed consent approved by the Medical Ethics Committee of the KU Leuven and were scheduled to take part in a baseline measurement. During baseline measurement, participants completed standardized measurements including height, weight, resting blood pressure, resting heart rate, waist and hip circumference, and measurement of fat percentage. Participants received the armband with oral and written instructions for proper wear and were given the opportunity to wear and adjust the armband. They were asked to wear the armband for 7 days to assess baseline physical activity level (bPAL). Participants received no feedback from the armband during this period. Participants were asked to fill in the Flemish Physical Activity Computerized Questionnaire (FPACQ; [45]) about their self-reported physical (in)activity pattern and were asked to complete some other questionnaires regarding their medical history, sports and activity history, and job status. The armband and the questionnaires were returned during their next visit. Previous research showed that both Saturday and Sunday and at least 3 weekdays needed to be monitored to obtain reliable measures of habitual physical (in)activity [46]. Only participants with a bPAL value lower than 1.71, wearing the armband for more than 95% of the time and had at least 3 days of monitoring from Monday through Friday and both Saturday and Sunday measured, were eligible to continue. 

### 2.4. Randomization

After successfully completing the baseline measurement and signing the informed consent, eligible participants were randomized into one of the four groups. During the randomization visit, all participants received an evaluation of their baseline measurement. The feedback report also gave information about the benefits of an active lifestyle, the current PA recommendations, how to get active in daily life, and tips for starting PA.

Table 1 shows the descriptive statistics of our study population. Our population includes participants with a bPAL ≥ 1.71 MET (Act), individuals that dropped out from the intervention trial (DO), and the four intervention arms (MIG, PG, DG, and CoachG). The randomization process achieves a reasonable balance across the four intervention arms at baseline. After exclusion of the active group and the drop-out group, post hoc tests reveal a difference in bPAL between the PG and DG of 0.08 MET for the total group and 0.11 MET for the male group. No difference in bPAL exists between the PG and DG for the female group. 

### 2.5. Followup Examinations

Followup data collection visits occurred at 3, 6, and 12 months after randomization and was collected between October 2010 and July 2012. The same measurements completed during the baseline session were assessed during each followup visit. Participants were also asked to fill in the FPACQ to assess detailed information on several dimensions of PA and sedentary behavior over a usual week. After each followup measurement, participants received feedback about their physical (in)activity level and a comparison was made with previous measurements.

### 2.6. Outcome Measurements and Methods

The Primary outcome measurement is the physical activity level (PAL). Secondary outcome measures include step count, minutes of physical (in)activity (sedentary, light, moderate, vigorous, and very vigorous intensity PA), daily energy expenditure in PA, percent of participants losing fat percentage, and stages of motivational readiness for PA. 

#### 2.6.1. Anthropometric and Physiological Measures

During each assessment, several measures were obtained including height, weight, hip, and waist circumference, resting blood pressure, resting heart rate, and fat percentage. Resting heart rate and resting blood pressure were measured alternately three times to improve measurement accuracy. Height and weight were assessed without shoes using a portable stadiometer of Martin (GPM anthropological instruments, Zurich, Switzerland) and a digital scale (Seca, Hamburg, Germany). BMI is calculated as body weight/height². The same measuring tape with tension rod was used for each waist and hip circumference measure. The waist was measured at the smallest circumference of the natural waist, just above the belly button. Hip circumference was assessed with the measuring tape being placed just above both iliac crests. Bioelectrical impedance analysis (BIA) was used to obtain a complete analysis of the body composition (percent distribution of body fat). All participants were measured by the same person.

#### 2.6.2. Physical Activity Assessment

Physical activity levels were assessed using the armband. The armband is a PA monitor that is worn on the upper left arm halfway between the acromion and olecranon processes. This device measures physical (in)activity using accelerometry technology augmented by two heat sensors (a thermistor-based skin surface sensor and a proprietary heat flux sensor) and a galvanic skin response sensor. These four internal sensors turn on the monitor (i.e., with skin contact) and estimate armband compliance, daily energy expenditure, step count, sleep efficiency, and the intensity, duration, and frequency of PA bouts. Collected PA data were downloaded during the baseline, 3-month, 6-month, and 12-month assessments. All armband data were analyzed by computer-based software (version 6.1) at 1-minute intervals using demographic information (i.e., gender, age, height, and weight at prior assessment) and proprietary algorithms. Throughout the study, the DG and CoachG group participants used the self-monitoring device to aid behavior change via real-time lifestyle feedback targeting PA.

 Baseline PA occurred over a one-year period (July 2010–July 2011) which makes it possible to account for seasonal variation in PA in a predominantly sedentary population.

#### 2.6.3. SenseWear Armband Validity

Independent researchers have validated the SenseWear system, especially with respect to comparing energy expenditure measurements from the Armband to estimates from gold-standard laboratory equipment [47–50]. An extensive review of the literature shows different validation studies where several SenseWear models (SenseWear, SenseWear pro, SenseWear pro2, and SenseWear pro3) and software algorithms are used. The SenseWear Armband has been validated as a measure of daily PA with findings suggesting that both minute-by-minutes as well as average energy expenditure can be a valid measurement of “free-living” activity [51, 52]. Previous validation studies have merely focused on low-to-moderate intensity activities. Two recent studies [53, 54] discussed the validation of the SWA at higher intensity levels and found a significant underestimation of the armband compared to indirect calorimetry for vigorous and very vigorous exercise intensities.

### 2.7. Statistical Analysis

Differences between the four study arms in PA outcomes will be tested according to the intention-to-treat philosophy. All participants' data will be analyzed according to their group assignment at randomization, regardless of adherence to the intervention. The PA outcomes being studied are daily energy expenditure (DEE), active energy expenditure (AEE), minutes of PA, physical activity level (PAL), and steps. A primary comparison is between the PA outcomes of groups who receive feedback (step group, display group and coaching group) and the group receiving no feedback (minimal intervention group). We hypothesize that getting feedback on the PA pattern will alter the PAL compared to having no information. Secondly, a comparison will be made on the difference in PA variables between participants who received feedback by means of the SWA display (display group) and participants who are only given information on their daily step count using a simple pedometer (step group). Since the SWA display gives more detailed information on different PA variables (minutes of PA, DEE, and steps) in contrast to the pedometer which only gives information on the daily step count, it is expected that people from the display group will have more benefits of the intervention compared to those belonging to the step group. Finally, the additional effect of a coach having weekly meetings with the participants will be evaluated. We hypothesize that the power of the interaction of a coach will result in an increase of the PA outcomes compared to subjects only receiving information by means of a technological instrument (display group and step group). Differences in PA outcome measures will be examined using analysis of covariance (ANCOVA) models of 12-month change scores. Self-reported PA, awareness, and cognitions will be analyzed as secondary outcomes. All analyses take into account specified covariates, including age, gender, BMI, education, percentage of armband wearing time, and baseline values of PA outcome measures. 

Based on our results from a pilot study (*n* = 52; PALpre = 1.41 ± 0.11 METs), an a priori power analysis was performed to determine the sample size necessary to detect a significant increase in energy expenditure in the intervention groups. Sample size calculations showed that 57 subjects in each group would give 80% power to detect a difference in PAL between baseline and posttest at a significance level of *P* < 0.05.

## 3. Intervention

### 3.1. Intervention Groups

All intervention groups receive a feedback report on the physical activity level, amount of steps taken, and minutes of physical (in)activity. In a personal contact with a coach, the report is discussed. The intervention study was executed by one coach to maximize the reliability of the intervention trial.

#### 3.1.1. Minimal Intervention Group

During the 4-week intervention period, this group (25 male, 31 female) wears the SenseWear Armband but gets no further feedback. 

#### 3.1.2. Pedometer Group

The pedometer group (26 male, 31 female) receives feedback on the daily step count by means of a pedometer (Yamax Digiwalker, SW200) and wears the SenseWear Armband for measuring the physical activity level. The Yamax SW Digiwalker is a waist-mounted device and is the most widely used pedometer in research studies. Previous studies comparing 10 or more pedometer models identified it as one of the most accurate and reliable electronic pedometers available [14]. Every day participants of the pedometer group write down their amount of steps in a step diary and also mention the reason for walking 10,000 steps a day. After an intervention period of 4 weeks, the pedometer is returned and participants are asked to wear the SenseWear Armband for one more week without knowing the amount of steps taken every day.

#### 3.1.3. Display Group

The display group (26 male, 31 female) receives a SenseWear display which allows participants to concurrently track total calories burned, minutes spent performing PA, and number of steps taken throughout the day. Participants wear the SenseWear Armband and matching SenseWear Display for a period of 4 weeks. Every day participants of the display group write down their amount of calories burned, their amount of steps taken, and their minutes of PA a day in an activity diary. They are asked to mention the reason for achieving or not succeeding in their personal goals. Every participant has individualized target goals for total calories, number of steps, and minutes spent performing PA. The individualized energy expenditure goal corresponds with an increase of the individual physical activity level with 0.10 MET. Individuals are asked to walk 10,000 steps a day and to be physically active for 60 minutes a day. After an intervention period of 4 weeks, the SenseWear Display is returned and participants are asked to wear the SenseWear Armband for one more week without knowing the amount of calories burned, amount of steps taken, or amount of minutes spent in PA every day.

#### 3.1.4. Coaching Group

The coaching group (26 male, 31 female) receives the same intervention as the display group (see above) but additionally has a weekly meeting with a coach to discuss the progress they have made. The session format is as follows: check-in of SenseWear data, discussion of results, evaluation of the individual goal setting, summary of current session, and preview of the next session.

## 4. Discussion

This intervention study will evaluate the effectiveness of four approaches to enhance PA in daily life over a one-year period in sedentary Flemish employees. The focus of this study is to evaluate the efficacy of providing real-time feedback on physical (in)activity patterns. Secondly our intervention study will investigate whether feedback on several parameters (including energy expenditure, steps, and minutes of physical (in)activity performed) will increase the physical activity level in comparison with a standard 10,000 step program. Our final aim is to evaluate whether a weekly meeting with a personal coach will help create an autonomous supportive environment and stimulate the feeling of relatedness thereby influencing the PA behavior. It should be noted that the participants in this study are volunteers and, as such, may be different (e.g., psychologically or motivationally) from other sedentary individuals. Therefore, results from this study need to be interpreted with caution. 

## Figures and Tables

**Table 1:** Baseline characteristics.

Characteristics	Total (*n* = 320)	Act (*n* = 82)	DO (*n* = 7)	MIG (*n* = 56)	PG (*n* = 57)	DG (*n* = 57)	CoachG (*n* = 57)
Age (years)	41.0 (10.7)	38.2 (10.0)	42.4 (16.3)	40.6 (11.1)	43.3 (10.7)	43.9 (10.3)	40.8 (9.8)
♂	43.0 (10.4)	38.3 (10.2)	59.0 (—)	44.8 (9.9)	45.6 (10.5)	46.0 (10.7)	42.0 (8.9)
*♀*	39.4 (10.6)	38.1 (10.0)	39.7 (16)	37.4 (11.1)	41.3 (10.7)	42.1 (9.8)	39.7 (10.5)
Weight (kg)	77.2 (15.7)	69.3 (14.6)	71.6 (12.0)	78.0 (13.4)	79.8 (15.7)	81.0 (14.6)	82.5 (17.1)
*♂*	87.5 (13.3)	80.4 (12.2)	95.3 (—)	86.4 (11.9)	91.4 (10.8)	88.2 (13.4)	94.2 (14.6)
*♀*	68.7 (12.1)	59.2 (7.5)	67.7 (6.4)	71.2 (10.5)	70.1 (12.2)	74.9 (12.8)	72.8 (12.3)
Height (cm)	171.3 (9.3)	170.5 (7.7)	163.9 (11.3)	171.8 (9.7)	172.5 (10.7)	171.4 (8.2)	171.9 (9.4)
*♂*	178.7 (6.6)	175.9 (5.4)	175.9 (—)	178.6 (8.2)	181.6 (7.1)	178.4 (6.5)	179.9 (4.7)
*♀*	165.2 (6.3)	165.6 (6.0)	161.9 (10.9)	166.4 (7.0)	164.8 (6.4)	165.6 (3.9)	165.2 (6.6)
BMI (kg/m²)	26.2 (4.2)	23.7 (3.7)	26.6 (2.9)	26.3 (3.3)	26.7 (4.2)	27.5 (3.9)	27.8 (4.4)
*♂*	27.4 (3.7)	26.0 (3.6)	30.8 (—)	27.0 (3.0)	27.7 (3.0)	27.6 (3.3)	29.1 (4.5)
* ♀*	25.2 (4.3)	21.6 (2.4)	25.9 (2.4)	25.7 (3.4)	25.9 (4.9)	27.3 (4.4)	26.7 (4.1)
WC (cm)	84.8 (12.4)	78.9 (12.0)	83.5 (9.7)	85.7 (10.5)	86.5 (12.5)	88.0 (11.2)	88.4 (13.6)
*♂*	92.6 (10.4)	88.0 (9.9)	103.5 (—)	91.2 (9.3)	94.6 (9.0)	93.8 (10.3)	97.4 (11.4)
*♀*	78.5 (10.1)	70.6 (6.4)	80.1 (4.2)	81.3 (9.4)	79.8 (10.9)	83.1 (9.6)	80.9 (10.4)
HC (cm)	103.4 (8.0)	98.4 (6.7)	105.2 (4.9)	103.2 (6.6)	105.0 (8.0)	106.2 (9.1)	106.2 (7.4)
*♂*	104.9 (6.9)	102.1 (5.9)	110.7 (—)	103.3 (7.0)	106.5 (5.8)	105.6 (8.1)	108.0 (6.4)
*♀*	102.2 (8.7)	95.0 (5.4)	104.3 (4.6)	103.2 (6.5)	103.8 (9.3)	106.7 (9.9)	104.6 (8.0)
Waist-hip ratio	0.82 (0.08)	0.80 (0.08)	0.79 (0.08)	0.83 (0.08)	0.82 (0.09)	0.83 (0.08)	0.83 (0.09)
*♂*	0.88 (0.07)	0.86 (0.07)	0.93 (—)	0.88 (0.06)	0.89 (0.06)	0.89 (0.07)	0.90 (0.07)
*♀*	0.77 (0.06)	0.74 (0.04)	0.77 (0.05)	0.79 (0.06)	0.77 (0.06)	0.78 (0.05)	0.77 (0.07)
SBD (mmHg)	122 (16)	119 (14)	112 (15)	122 (16)	127 (17)	125 (17)	121 (15)
*♂*	130 (14)	126 (12)	144 (—)	128 (15)	136 (13)	130 (14)	129 (14)
*♀*	116 (15)	112 (12)	107 (7)	117 (16)	119 (17)	122 (18)	113 (11)
DBD (mmHg)	80 (10)	78 (9)	72 (12)	82 (10)	83 (11)	83 (11)	78 (9)
*♂*	83 (10)	81 (8)	99 (—)	83 (10)	87 (11)	85 (10)	81 (9)
*♀*	78 (10)	74 (9)	68 (5)	81 (11)	80 (10)	82 (11)	76 (7)
HR (bpm)	71 (11)	68 (11)	77 (11)	72 (12)	72 (11)	71 (12)	71 (9)
*♂*	70 (11)	68 (12)	71 (—)	71 (13)	69 (10)	69 (11)	72 (10)
*♀*	72 (11)	68 (11)	78 (12)	73 (11)	75 (10)	73 (12)	69 (9)
% Body fat (*n* = 319)	27.6 (6.8)	22.9 (5.5)	32.6 (4.6)	28.4 (6.6)	29.3 (6.6)	29.6 (6.9)	29.3 (5.8)
*♂*	24.6 (5.9)	21.6 (6.1)	31.5 (—)	24.7 (6.1)	25.9 (5.1)	25.3 (5.2)	26.9 (5.0)
*♀*	30.1 (6.5)	24.0 (4.8)	32.8 (5.0)	31.6 (5.3)	32.1 (6.4)	33.3 (6.0)	31.2 (5.8)
bPAL (MET) (*n* = 316)	1.55 (0.26)	1.88 (0.18)	1.51 (0.16)	1.46 (0.15)	1.39 (0.16)	1.47 (0.16)	1.41 (0.17)
*♂*	1.55 (0.25)	1.87 (0.17)	1.19 (—)	1.44 (0.14)	1.38 (0.13)	1.49 (0.12)	1.41 (0.16)
*♀*	1.55 (0.26)	1.89 (0.19)	1.56 (0.08)	1.47 (0.15)	1.40 (0.18)	1.45 (0.19)	1.40 (0.18)

Abbreviations: Act: participants with bPAL ≥ 1.71 MET; DO: drop out from study; MIG: minimal intervention group; PG: pedometer group; DG: display group; CoachG: coaching group; WC: waist circumference; HC: Hip circumference; SBD: systolic blood pressure; DBD: diastolic blood pressure; HR: mean heart rate; bPAL: physical activity level at baseline; MET: metabolic equivalent.

## Abbreviations

bPAL: Physical activity level at baseline
MET: Metabolic equivalent.
